# PARTIAL HEPATECTOMY USING LINEAR CUTTER STAPLER: ARE THERE ADVANTAGES?

**DOI:** 10.1590/0102-672020230057e1775

**Published:** 2023-12-08

**Authors:** Marcio Fernandes CHEDID, Pietro Waltrick BRUM, Tomaz de Jesus Maria GREZZANA-FILHO, Rafaela Kathrine da SILVA, Pedro Funari PEREIRA, Aljamir Duarte CHEDID, Cleber Rosito Pinto KRUEL

**Affiliations:** 1 Universidade Federal do Rio Grande do Sul, Porto Alegre University Hospital, Hepatobiliary Surgery and Liver Transplantation Unit – Porto Alegre (RS), Brazil.

**Keywords:** Liver, Tumors, Hepatectomy, Surgical staplers, Fígado, Tumores, Hepatectomia, Grampeadores cirúrgicos

## Abstract

**BACKGROUND:**

Morbidity of liver resections is related to intraoperative bleeding and postoperative biliary fistulas. The Endo-GIA stapler (EG) in liver resections is well established, but its cost is high, limiting its use. The linear cutting stapler (LCS) is a lower cost device.

**AIMS:**

To report open liver resections, using LCS for transection of the liver parenchyma and *en bloc* stapling of vessels and bile ducts.

**METHODS:**

Ten patients were included in the study. Four patients with severe abdominal pain had benign liver tumors (three adenomas and one focal nodular hyperplasia). Among the remaining six patients, four underwent liver resection for the treatment of colorectal liver metastases, three of which had undergone preoperative chemotherapy. The other two cases were one patient with metastasis from a testicular teratoma and the other with metastasis from a gastrointestinal neuroectodermal tumor.

**RESULTS:**

The average length of stay was five days (range 4–7 days). Of the seven patients who underwent resections of segments II/III, two presented postoperative complications: one developed a seroma and the other a collection of abdominal fluid who underwent percutaneous drainage, antibiotic therapy, and blood transfusion. Furthermore, the three patients who underwent major resections had postoperative complications: two developed anemia and received blood transfusions and one had biloma and underwent percutaneous drainage and antibiotic therapy.

**CONCLUSIONS:**

The use of the linear stapler in hepatectomies was efficient and at lower costs, making it suitable for use whenever EG is not available. The size of the LCS stapler shaft is more suitable for *en bloc* transection of the left lateral segment of the liver, which is thinner than the right one. Further studies are needed to evaluate the safety of LCS for large liver resections and resections of tumors located in the right hepatic lobe.

## RESUMO

**RACIONAL:**

A morbidade das ressecções hepáticas está relacionada a sangramento intraoperatório e fístulas biliares pós-operatórias. O grampeador Endo-GIA (EG) em ressecções hepáticas está bem estabelecido, mas o seu custo é elevado, limitando seu uso. O grampeador de corte linear (LCS) é um dispositivo com menor custo.

**OBJETIVOS:**

Relatar ressecções hepáticas abertas, empregando o LCS para transecção do parênquima hepático e grampeamento em bloco de vasos e ductos biliares.

**MÉTODOS:**

Dez pacientes foram incluídos no estudo. Quatro pacientes com dor abdominal importante apresentavam tumores hepáticos benignos (três adenomas e um hiperplasia nodular focal). Dentre os demais seis pacientes, quatro foram submetidos à ressecção hepática para o tratamento de metástases hepáticas colorretais, sendo que três deles haviam sido submetidos à quimioterapia pré-operatória. Os dois outros casos foram um paciente com metástase de teratoma testicular e o outro com metástase de tumor neuroectodérmico gastrointestinal.

**RESULTADOS:**

O tempo médio de internação foi de 5 dias (variação=4-7 dias). Dos sete pacientes submetidos a ressecções dos segmentos II/III, dois apresentaram complicações pós-operatórias: um paciente desenvolveu seroma e o outro uma coleção de fluido abdominal submetido a drenagem percutânea, antibioticoterapia e transfusão de sangue. Além disso, os três pacientes submetidos a ressecções maiores tiveram complicações pós-operatórias: dois pacientes desenvolveram anemia e receberam transfusões de sangue e um paciente apresentou biloma e foi submetido a drenagem percutânea e antibioticoterapia.

**CONCLUSÕES:**

O emprego do grampeador linear nas hepatectomias foi eficiente e a custos mais baixos, tornando-o adequado para uso sempre que EG não estiver disponível O tamanho da haste do grampeador LCS é mais adequado para a transecção em bloco do segmento lateral esquerdo do fígado, que é mais fino que o direito. Novos estudos são necessários para avaliar a segurança do LCS para grandes ressecções hepáticas e ressecções de tumores localizados no lobo hepático direito.

## INTRODUCTION

Liver resections are traditionally associated with significant morbidity and mortality, most notably related to intraoperative blood loss and postoperative biliary leaks^4^. Technological advances have provided an armamentarium of hemostatic devices that can be utilized during liver resections. Parenchymal liver division can be performed by several means, including crush clamp parenchymal fracture or ultrasonic aspiration with the tying and/or clipping of blood vessels and bile ducts. The use of vascular staplers Endo-GIA (EG) for safely securing and dividing inflow and outflow vessels in major liver resections is described in the literature and is well-established^7^.

Regarding the use of staplers for parenchymal division, almost all studies reported on the use of EG^2,12-14^ and/or thoracoabdominal (TA) stapler^6,8,11,16^. TA staples vessels but does not divide them. The cost of EG varies from country to country and may be considerably high in some developing countries, limiting its utilization.

The linear cutting stapler (LCS) (75 mm linear cutter, Ethicon, Raritan, New Jersey, USA^13^ is the only prior evidence of LCS use in liver resections in humans. The present study reports on a series of liver resections in which LCS was employed both for vessel stapling and parenchymal transection in an *en bloc* fashion. Besides analyzing the outcomes, a cost comparison between the endoscopic and the linear device was also outlined in this report.

## METHODS

All consecutive open liver resections employing LCS technique performed from July 2017 to November 2018 were included. A total of ten patients were studied (Table 1). Six patients were female (including four benign cases) and two were male. The median age was 43.5 years (range 25–67). The study received approval from the Institutional Board Review Committee of the Porto Alegre University Hospital (RS) (number 2017-0255).

**Table 1:** Data of ten consecutive patients undergoing liver resection using linear cutting stapler.

Nº	Age	Sex	Race	Histology	Preop Chemo	Resection subtype	Additional procedure	Cartridge number	Time	Margins	Complication	Status	Bleeding	Pringle
1	26	F	W	Adenoma	-	Segment III	N	1	90	Free	No	Alive	150	N
2	50	F	W	Adenoma	-	Segments II, III	N	2	203	Free	No	Alive	150	Y
3	42	F	B	FNH	-	Segment III	N	3	65	Free	Seroma	Alive	388	Y
4	39	F	W	Adenoma	-	Segments II, III	N	1	144	Free	No	Alive	900	Y
5	64	F	W	CLm	Y	Right lobe	Y	3	328	Free	Blood transfusion	Dead	1,300	N
6	66	F	W	CLm	N	Segments II, III	N	2	226	Free	Collection; blood transfusion	Dead	1,140	N
7	45	M	W	CLm	Y	Segments II, III	Y	2	265	Free	No	Alive	758	N
8	67	M	W	CLm	Y	Segment II	Y	2	410	Free	No	Alive	150	Y
9	25	M	W	TTm	Y	Right lobe	N	1	310	Free	Bilioma	Alive	1,500	Y
10	38	M	W	GNETm	Y	Segments VI,VII	Y	3	440	Free	RBC transfusion	Alive	2,325	Y

Preop Chem: preoperative chemotherapy; F: female; M: male; W: white; B: black; Y: Yes; N: No; FNH: focal nodular hyperplasia; CLm: colorectal metastasis; TTm: teratoma testicular; GNETm: gastrointestinal neuroectodermal metastasis; RBC: red blood cell.

### Surgical technique

The LCS was initially applied to liver resections of tumors located in segments II/III. Anatomic left lateral hepatectomy (LLHP) was employed for larger tumors, whereas partial non-anatomic LLHP was employed for the smaller ones. Progressively, LCS was also used in resections of tumors located in liver segments other than II/III, including major hepatectomies such as in the right liver lobe. The LCS staple height was 3.5 mm (blue color).

Data extracted from the medical records of the study patients included demographic variables, diagnosis and indication for liver resection, type of liver resection, and previous procedures. Perioperative variables were recorded such as operative time, number of stapler loads used, operative blood loss, need for blood transfusion, and whether portal occlusion was performed. Postoperative variables included length of stay, margin status, reoperations, and complications classified according to Clavien-Dindo scale^1^.

**Figure 1:**
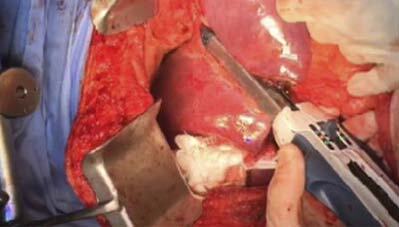
Stapler closure for transection of the liver parenchyma and en bloc cutting and stapling of liver vessels and bile ducts for complete anatomic left lateral hepatic sectionectomy.

**Figure 2:**
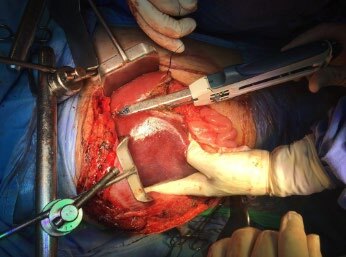
Second staple firing for transection of the liver parenchyma and *en bloc* cutting and stapling of liver vessels and bile ducts for complete anatomic left lateral hepatic sectionectomy.

**Figure 3:**
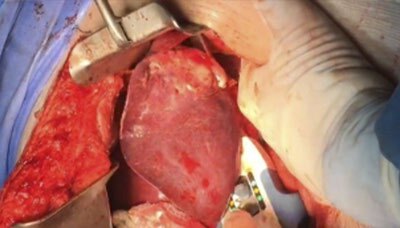
Third staple firing for transection of the liver parenchyma and en bloc cutting and stapling of liver vessels and bile ducts for complete anatomic left lateral hepatic sectionectomy (liver tumor located on the left liver edge).

**Figure 4:**
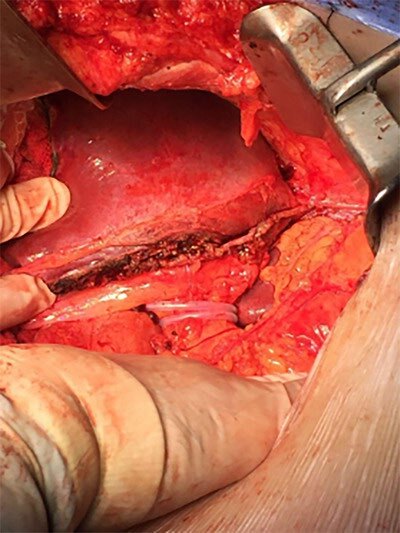
Final aspect of edge liver surface close to falciform ligament (after complete left lateral hepatic sectionectomy).

The LLHP was performed preferentially using an open midline or a J-shaped incision (whenever feasible). The ‘Mercedes’ incision was reserved for technically demanding cases (e.g., obese patients, larger tumors). The ipsilateral triangular ligament was taken down and the surface of the liver was completely exposed. Hepatogastric ligament was incised and the porta hepatis was controlled. Pringle maneuver was generally avoided, being performed in cases of bleeding^15^. For total LLHP, the lesser omentum was not opened, and the Arantius duct was not divided. Parenchymal transection line was set on the line about 1 cm away from the left side of the falciform ligament. Parenchymal division was accomplished by cutting the overlying Glisson’s capsule and the first two centimeters of liver parenchyma’s depth (on both anterior and posterior liver surfaces) with electrocautery, from the ventral to the dorsal side and from the caudal to the cranial side, without exposing the main trunk of the left hepatic vein or the portal pedicles for segments II and III. The narrow ends of LCS were then passed through the open surface and closed, compressing the parenchyma for at least 30 seconds (Figure 1). Thus, the stapler was fired, cutting the liver parenchyma and cutting and stapling the liver vessels and bile ducts in an *en bloc* fashion (Figures 2 and 3). Usually, one or two additional stapling firings were necessary for the complete liver transection. The portal pedicles for segments II and III were divided *en bloc* by the stapling firings. Generally, the last staple firing was directed 3 to 4 cm away from the falciform ligament in the cranial part of the resection. Thus, the branches of the left hepatic vein (rather than the vein itself) were divided using the LCS. The raw liver surface was then inspected and oozing vessels and biliary ducts were controlled with needle sutures whenever necessary (Figure 4). A J-Blake drain was positioned close to the liver surface right before abdomen closure.

## RESULTS

Four out of the total ten patients had symptomatic benign liver tumors (three adenomas and one focal nodular hyperplasia [FNH]). All these four patients had abdominal pain as the main indication for liver resection. Of the remaining six patients, four were submitted to liver resection for the treatment of colorectal liver metastases (CRLm), of which three underwent preoperative chemotherapy. The remaining two cases included one patient with metastasis from testicular teratoma (TTm) and another with gastrointestinal neuroectodermal metastasis (GNETm). 

Overall, seven LLHP (four complete and three partial) were performed. The remaining three cases in the present series comprised two right hemi-hepatectomies and one segmentectomy of VI/VII (right lateral hepatic sectionectomy). A median of two stapler loads was used per surgery. In nine out of the ten cases, up to three loads were employed (in the remaining case, seven loads were used in a right hemi-hepatectomy in order to accelerate the liver parenchyma transection).

For the seven LLHP, the median operative time was 203 minutes (range 65–410), and the median blood loss was 388 cc (range 150–1,140). In two of these seven minor liver resections, additional liver procedures were performed during the same operative time (nodulectomies in segments V/VII and III/IVb). For the five cases in which no additional procedures other than LLHP were performed, the median operative time was 144 minutes (range 65–226).

For the three remaining liver resections in this series (two right hemi-hepatectomies and one right lateral hepatectomy), the median operative time was 328 minutes (range 310–440) and the median blood loss was 1,500 cc (range 1,300–2,325). Blood transfusion was performed in two of those three major liver resections.

All ten patients included in this series were discharged home safely before the end of the first postoperative week. The mean hospital stay was five days (range 4–7). Of the seven patients who underwent II/III resections, two experienced postoperative complications: one patient presented a seroma (Clavien-Dindo Grade 1), and one presented abdominal fluid collection (Clavien-Dindo Grade 3) and was submitted to percutaneous drainage and antibiotic therapy, also requiring a blood transfusion. Besides, all the three patients submitted to major resections experienced some postoperative complications as well: two required blood transfusions (Clavien-Dindo Grade 2) and one developed a bilioma and underwent percutaneous drainage and antibiotic therapy (Clavien-Dindo Grade 3)^1^.

In all ten cases, postoperative histopathological studies revealed free margins. During colorectal metastasis (CLm) follow-up, two of the four patients died from tumor liver failure, 15 and 34 months after surgery. Two other patients lost follow-up, 6 and 33 months after the surgery. The remaining two patients who underwent liver resection of malignant tumors, other than CLm, are currently alive. The four patients who underwent liver resection for benign tumors (one FNH and three adenomas) are alive and being followed in an outpatient unit.

## DISCUSSION

This study analyzed ten consecutive open liver resections using LCS for *en bloc* liver transection and stapling of minor liver vessels and biliary ducts. Seven of the cases comprised total or partial LLHP. Complete LLHPs were performed with LCS in an anatomic fashion, and partial LLHPs were removed in a non-anatomic fashion^3^. LCS was also employed in three patients undergoing major liver resections (two right hepatectomies and one right posterior hepatic sectionectomy [RPHS]) in order to expedite liver transection. RPHS was included as a major resection because it encompasses a potential for complications similar to that of major liver resections.

Analyzing the literature on liver resections employing staplers, almost all prior studies included EG and/or TA staplers^5^. The authors found just one previous study with only two cases of liver resections employing LCS in humans^3^. Thus, our study adds a relevant piece of information about the safety of LCS in liver resection in humans.

Stapler use offers several advantages to liver resections: ease of use, rapidity of tissue division, and *en bloc* closure of both vascular and biliary structures. In a randomized clinical trial, stapler hepatectomy proved to be non-inferior to the traditional clamp-crushing technique^9^. The largest retrospective cohort on endoscopic stapler hepatic parenchymal resection included 1,174 cases^10^. Compared to this study, ours included a younger patient profile (median age 43.5 years vs. 56 years). Opposed to that study, a majority of female patients (60 vs. 48%) was demonstrated in our series. Since benign liver tumors are known to be more common in women, such preponderance can be justified by the 40% of benign etiology in our series (vs. 3.5% in that literature study). Even so, in both studies most resections included malignant tumors, and colorectal metastasis was the most common subtype of malignancy.

Our study revealed zero mortality for a total of ten consecutive patients who underwent liver resection employing LCS. For the resection of liver segments II/III, a 28.5% complication rate was recorded in our series, with outcomes corroborating those in the literature^10,14^. Concerning other perioperative outcomes, such as median blood loss, operative time, and blood transfusion requirement as an isolated complication (Clavien-Dindo Grade 3)^1^, our results with minor resection achieved similar outcomes to those of the subset of patients undergoing minor liver resection using EG in a large study^14^.

EG is considered the ideal stapler for performing both open and laparoscopic liver resections. However, due to its price, the use of EG may be limited in some scenarios, especially in developing countries. Most of the hospitals in the Public Brazilian Health System (SUS), for instance, is not currently using EG. As of January 2023, the price of the EG is 170 USD in Brazil, and each additional load costs 140 USD. Ethicon LCS (Ethicon, Raritan, New Jersey, USA^10^ revealed an average number of seven stapler loads. Thus, performing a liver resection with the EG stapler (with six additional loads) in Brazil would cost 1,010 USD vs. 192 USD for the same operation with LCS (only one additional load is necessary on average). Moreover, the number of loads may not be completely predictable, and the price of additional loads for EG is 2.9 times more expensive than the LCS one, which may outstandingly raise the cost of EG stapler to perform a liver resection.

One limitation of the present study is its small sample size (n=10). In addition, most cases (seven of ten) included minor liver resections of tumors located in liver segments II/III (partial or complete LLHS). Besides, no control group was included in the study. One should also take into account that, in the seven LLHS cases, no vascular injuries or significant bleeding occurred with the use of LCS for *en bloc* transection of the liver parenchyma and staple closure of liver vessels.

## CONCLUSIONS

The usage of LCS for open minor liver resections of tumors located in liver segments II/III (LLHS) seems to be a safe practice. The size of the stapler steps of LCS (larger than the steps of EG) may be adequate for *en bloc* transection of the left liver lateral segment which is thinner than the right liver lobe. Thus, whenever EG is not available, LCS may be an option for partial or total LLHS. Also, LCS is associated with lower costs than EG, making it suitable for use in developing countries. Further studies are necessary to evaluate the safety of LCS for major liver resections and resections of tumors located in the right liver lobe. Furthermore, more evidence would be necessary to compare the efficacy and safety of LCS with the gold standard EG in liver resections.
